# McGirr on Abdominal Movements

**Published:** 1848

**Authors:** John E. McGirr

**Affiliations:** Professor of Anatomy, Physiology, and of Chemistry, in the University of the Lake, Chicago


### ARTICLE IV.

*Observations on Abdominal movements as connected with Pregnancy.* By John E. McGirr, A.M., M.D., Professor of Anatomy, Physiology, and of Chemistry, in the University of the Lake, Chicago.

In January last, Mrs. M., Æt. 41, applied to me for relief from the annoying symptoms that troubled her in this her eighth pregnancy. She considered herself now in her seventh month, and she had felt the motion of the child, she said, from about the usual period. At the beginning of each month, she was troubled with very severe lumber pains, and she sometimes feared a miscarriage; at each of these times a "small appearance," as she termed it, taking place, she was tolerably well until the next term. She remarked that she felt somewhat uneasy about her condition; that she did not think she had ever been subject to the same kind of sensations in any of her former pregnancies, and besides that, she was much smaller than she thought she ought to be; still, that the movements were so distinct as to leave no doubt on her mind as to her condition. But experience had long led me to doubt that because abdominal movements were present, therefore pregnancy must exist; and auscultation satisfied me that no young heart throbbed beneath her own. It is not necessary to pursue this case in detail—suffice to say that, in spite of her incredulity, the ninth, tenth, and eleventh months passed, and yet no "bairn" appeared to gladden its mother's heart; but that, now the uterine function is performed regularly, and she admits that "Doctors do know some things better than women!!"

The very remarkable and interesting case published in the last No. of the "Illinois and Indiana Medical and Surgical Journal," p. 554, under the heading "Abdominal Tumor mistaken for Pregnancy," is another instance in point. Here a distinguished practitioner was in error. "A movement like that of a living fœtus was distinctly felt;" "at five months the patient was conscious of a movement and pulsation in the abdomen, and believed herself pregnant." "The gradual increase of size went on, and the mother (who now slept with her daughter) said that the movement of the child continued. The patient complained of its violence in bed," &c. The lady died, and a post mortem examination revealed the nature of the case.

In 1844 I attended a lady, Mrs. R., Æt. 23, in her second pregnancy. Her case was a very tedious one; but as she said that she had constantly felt the motion of the child, there seemed to be no cause for interferance; but, after several hours of excruciating suffering, the powers of the mother began to flag. The os uteri being fully dilated, ergot was administered and the child was speedily born, but in so *putrid* a condition that it could scarcely be removed entire from the couch. She now told me that, sometime before, her little daughter had kicked her violently in the abdomen, so much so as to wake her from a deep sleep; that from that time until now she had suffered much. She expressed herself as having felt a "dead weight" in the abdomen, but, *as she felt the motion of her infant even more than before, she did not suspect its death.*

Instances the reverse of this, in which the child was born alive after all motion had ceased, and when the mother believed it dead, have occurred to every accoucheur who has attended many cases. It will not, therefore, be necessary to notice any one particularly.

If what I have said is correct, are operations, on the chances of which the lives of two human beings hang, to depend upon the absence or presence of these motions? "These movements are almost entirely attributed to the fœtus; there is hardly a point in utero-gestation upon which greater unanimity prevails"—and yet cases without number could be adduced which prove how fallacious is this test of pregnancy, or of the life or death of the fœtus, and which demonstrate, beyond the shadow of a doubt that the sensations of the mother and the manipulations of the practitioner are no safe guides, but that, if followed as they unfortunately too frequently are, they lead to the most lamentable and reprehensible results. How, I may be asked are we then to ascertain the existence of pregnancy, or the propriety of operating in doubtful cases if the presence or absence of these movements affords no indication? I answer that auscultation will afford the only reliable means of judging. In what a predicament then are those professing accoucheurs who refuse to avail themselves of this means of diagnosis, or who deny its advantages and unfortunately there are many, very many, who call the use of the stethoscope a humbug! If I could remove the covering which the grave places between them and their unfortunate victims, rashly, villanously, sacrificed, if I could hold up before their eyes their gory remains, streaming with blood, all of which might have been avoided by the use of this instrument, their slumbers would be haunted by more ghosts than ever visited the pillow of many a being who has paid, upon the scaffold, the penalty which the laws have imposed upon the shedder of the blood of his fellow man.

As this communication is already long enough, I will not lengthen it by farther observations that I intended to have made upon the cause and nature of these abdominal movements. These will form the subject of another paper.

